# Bilateral Glenohumeral-Acromioclavicular Composite Articulations Identified During Routine Cadaveric Dissection: A Case Report

**DOI:** 10.7759/cureus.115933

**Published:** 2026-09-08

**Authors:** Daniel Coovert, Hannah Knouse, Nathan Wrzesinski

**Affiliations:** 1 Biomedical Sciences, Northeast Ohio Medical University, Rootstown, USA; 2 Physical Therapy, ATI Physical Therapy, Indianapolis, USA; 3 Physical Therapy, Rockey Mountain Rehab Physical Therapy, Colorado Springs, USA

**Keywords:** acromioclavicular joint, acromion, anatomical variation, cadaveric dissection, clavicle, composite articulation, glenohumeral joint

## Abstract

The acromion and lateral clavicle normally lie outside the glenohumeral joint capsule. During routine dissection of an 80-year-old female donor, bilateral glenohumeral-acromioclavicular composite articulations were identified. In each shoulder, the surface opposing the humeral head extended superiorly to include a variant acromion and lateral clavicle; the humeral heads appeared morphologically typical. In the more extensively dissected right shoulder, the lateral end of the clavicle was broad, flattened, and polished where it opposed the humeral head, with a gross appearance consistent with chronic repetitive contact; no clearly defined articular cartilage was apparent. The infraspinatus and teres minor retained their normal humeral attachments, while variant fibers from both muscles blended with the expanded posterior capsule. The supraspinatus passed through an osseous tunnel formed by the variant acromial process and adjacent superior glenoid region before reaching its humeral insertion. The tendon of the long head of the biceps brachii was absent from the joint cavity and appeared to originate within the intertubercular sulcus. Morphometric analysis demonstrated increased acromial dimensions, acromioglenoid distance, and glenoid anteroposterior diameter compared to reference data. These bilateral composite articulations may have altered shoulder stability, range of motion, and rotator cuff mechanics. Recognition of this configuration may help distinguish anatomical variation from acquired shoulder disease.

## Introduction

The glenohumeral joint is normally formed by articulation between the head of the humerus and the glenoid cavity of the scapula. The relatively shallow glenoid cavity is deepened by the glenoid labrum, while the joint capsule, glenohumeral ligaments, and rotator-cuff tendons contribute to joint stability [1].

The acromion and lateral end of the clavicle form part of the superior osseous framework of the shoulder but do not normally contribute directly to the articular surface of the glenohumeral joint. The acromioclavicular and glenohumeral joints are typically separate synovial articulations enclosed by distinct joint capsules. Morphological variation of the scapula and acromion is well documented, but direct incorporation of the acromion and lateral clavicle into the glenohumeral articulation is not a typical reported configuration [2].

Acromial variation includes differences in shape, dimensions, slope, and the presence of an os acromiale [2]. Acquired secondary contact may also occur: full-thickness rotator-cuff tears can permit direct contact between the greater tubercle and the inferior acromion, producing an acromial grinding facet (facies articularis acromialis) [3]. In advanced cuff-tear arthropathy, the glenohumeral and acromioclavicular joints may communicate through a rotator-cuff defect and the subacromial bursa, producing the "geyser sign" [4]. This acquired communication differs from the present configuration, in which the variant acromion and lateral clavicle formed components of the concave surface opposing the humeral head within an expanded capsular space. A focused review of the literature did not identify a directly comparable bilateral composite articulation.

Variations that alter the relationship among the humeral head, glenoid cavity, acromion, and clavicle may influence shoulder mechanics and may be important during anatomical dissection, diagnostic imaging, arthroscopy, and surgical procedures. This report describes bilateral glenohumeral-acromioclavicular composite articulations in which the surfaces opposing the humeral heads included the glenoid cavity, a variant acromion, and the acromial end of the clavicle. The report emphasizes the gross morphology, capsular distribution, morphometric findings, and possible, though unproven, developmental basis of this configuration.

Portions of this case were previously presented as a poster at the Indiana State Doctor of Physical Therapy Capstone Research Event on May 27, 2019.

## Case presentation

During routine anatomical dissection by first-year Doctor of Physical Therapy students, bilateral glenohumeral-acromioclavicular composite articulations were identified in an 80-year-old female donor. Gross inspection demonstrated the same general composite configuration in the left shoulder; however, the left side was not dissected, measured, or photographed in sufficient detail for an equivalent morphometric or figure-based comparison. Accordingly, bilateral involvement was confirmed grossly, but detailed side-to-side comparison was not possible. The right shoulder was subsequently dissected and documented in greater detail.

Gross dissection of the right shoulder demonstrated an enlarged articulation with a prominent subacromial bursa occupying the space between the acromion, supraspinatus, and clavicle (Figure 1A). The acromion was elongated and possessed an additional osseous process that extended inferiorly toward the superior glenoid region. This process nearly fused with the superior glenoid region, forming an osseous arch and a distinct tunnel through which the supraspinatus muscle and tendon passed (Figure 1B).

**Figure 1:**
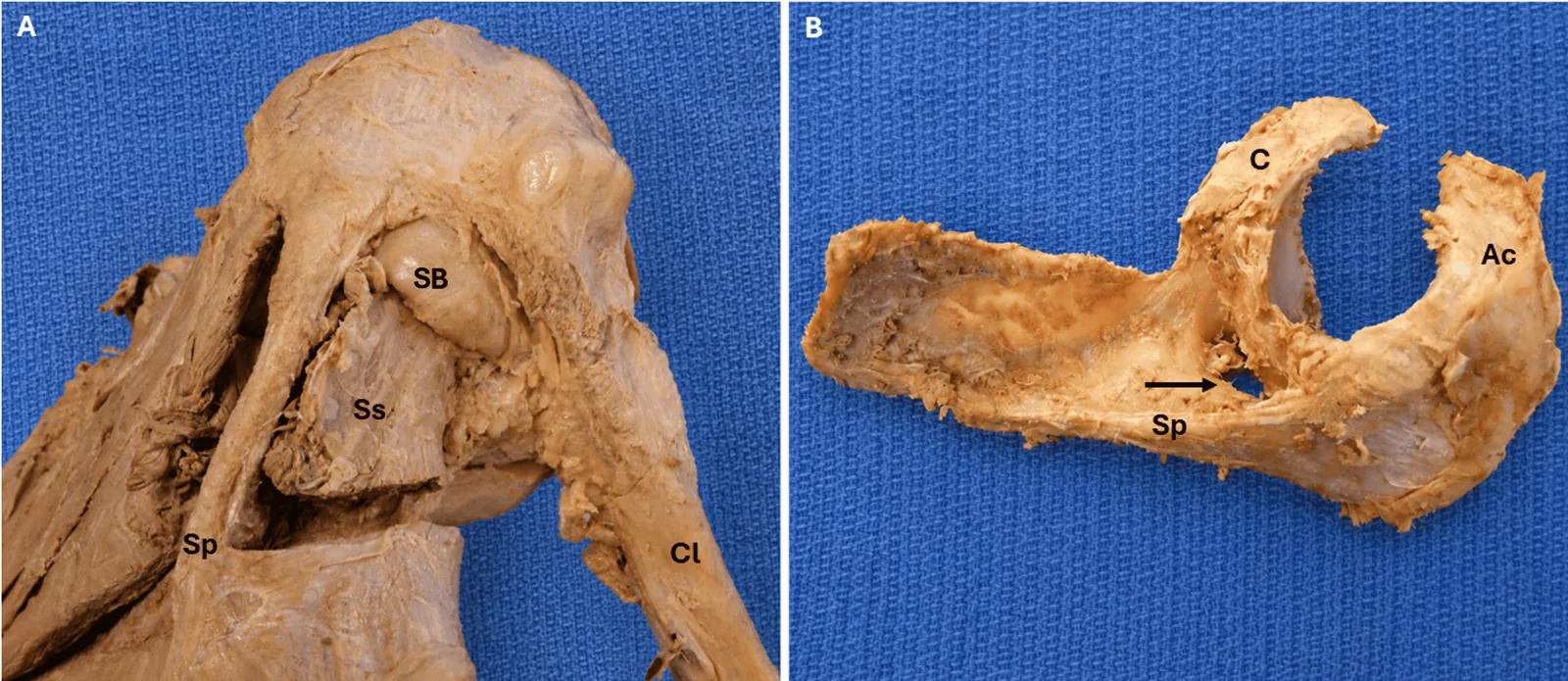
Relationship of the supraspinatus to the variant superior shoulder architecture. (A) Lateral view of the dissected right shoulder showing the supraspinatus (SS) passing beneath the abnormal superior osseous arch. The subacromial bursa (SB), scapular spine (Sp), and clavicle (Cl) are indicated; (B) Corresponding osseous specimen after soft-tissue removal demonstrating the tunnel formed by the variant acromial process and adjacent superior glenoid region (arrow), through which the supraspinatus muscle and tendon passed. The scapular spine (Sp), acromion (Ac), and coracoid process (C) are labeled; a smooth, articular-appearing surface extends anteriorly toward the coracoid.

Internally, rather than the glenoid cavity acting as the sole surface opposing the humeral head, the shoulder contained an expanded composite articulation. The right humeral head appeared morphologically typical, whereas the opposing concave surface included adjacent glenoid, acromial, and lateral clavicular components. Although these three components collectively formed a composite concave articulation opposing the humeral head, the tissue covering the components was not continuous; gross interruptions were visible at the transitions between surfaces. Osteophytes were present along the inferior glenoid rim adjacent to the labrum, and proliferative soft tissue was grossly apparent within the joint (Figure 2). Because histological examination was not performed, its tissue type and whether it represented pannus could not be established.

**Figure 2:**
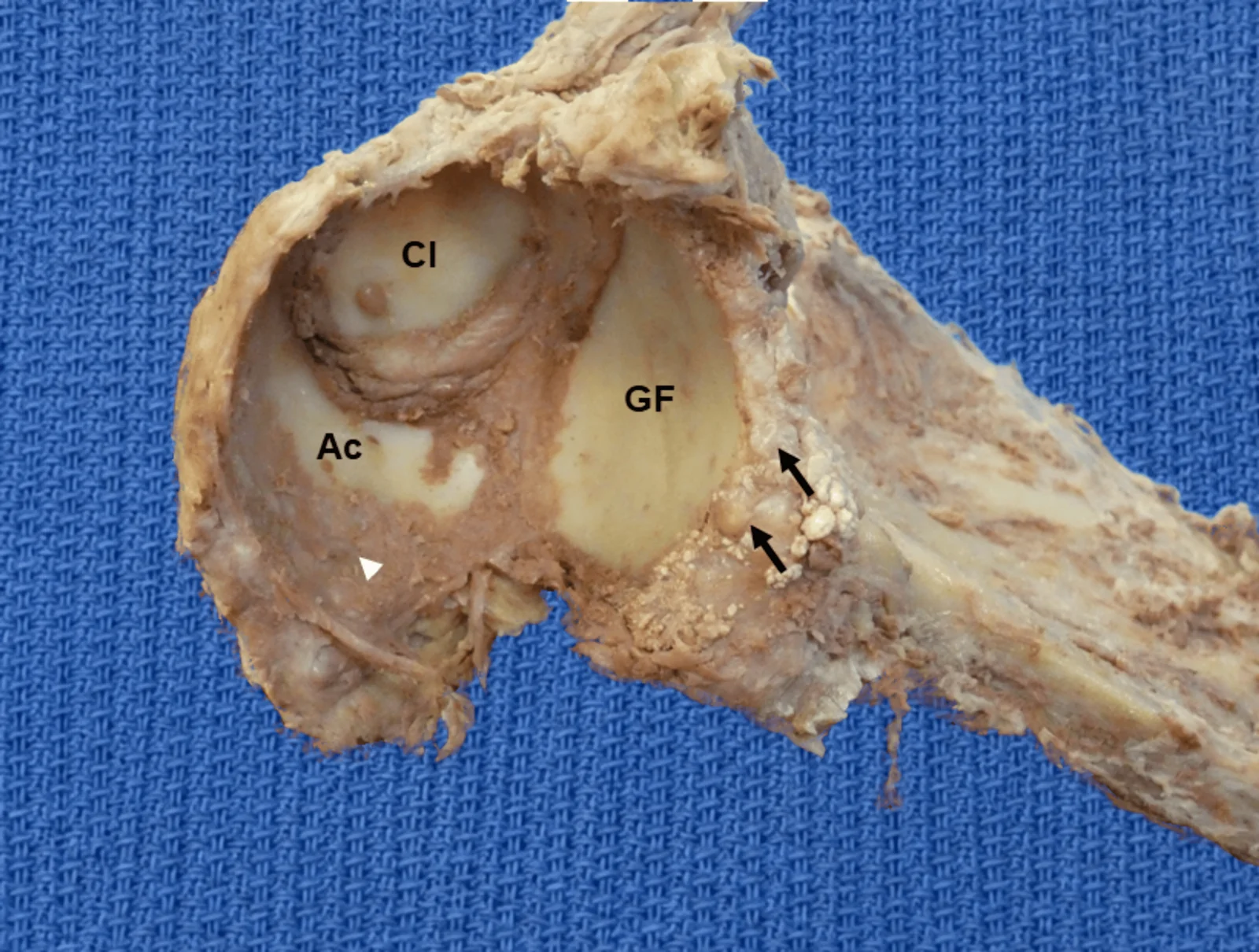
Internal view of the opened variant right glenohumeral joint demonstrating the composite concave surface that opposed the humeral head. The surface includes the glenoid cavity or glenoid fossa (GF), variant acromial component (Ac), and lateral clavicular component (Cl). Osteophytes (black arrows) and proliferative soft tissue (white arrowhead) are visible. These findings were interpreted as grossly consistent with superimposed degenerative joint changes; without histological examination, the soft tissue cannot be characterized definitively as pannus.

Compared with a typical clavicle, the lateral end of the variant clavicle was broad and flattened and exhibited a smooth, polished osseous surface where it was positioned to oppose the humeral head (Figure 3). This appearance was grossly consistent with eburnation, but histological examination was not performed; therefore, eburnation and the presence or absence of an articular cartilage remnant could not be confirmed.

**Figure 3:**
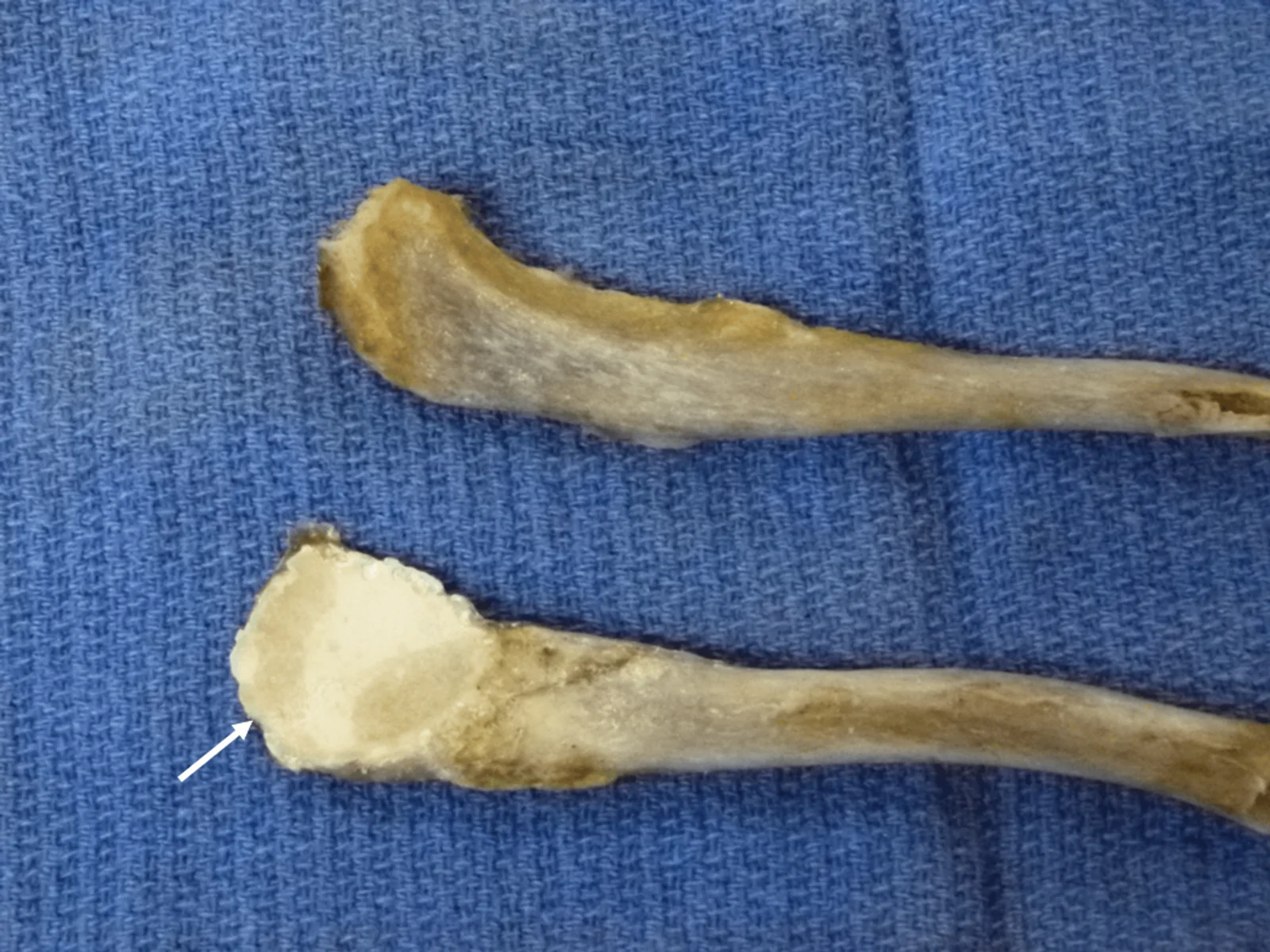
Comparison of the inferior acromial (lateral) ends of a typical clavicle (top) and the variant right clavicle (bottom). The arrow identifies the smooth, polished osseous surface of the variant lateral clavicle that was positioned to oppose the humeral head; the gross appearance was consistent with eburnation.

The glenoid cavity retained a generally typical shape but was enlarged relative to that of a typical scapula (Figures 4A, 4B). Comparison of the osseous specimens further demonstrated expansion of the variant acromion and glenoid-adjacent articular regions (Figures 4C, 4D).

**Figure 4:**
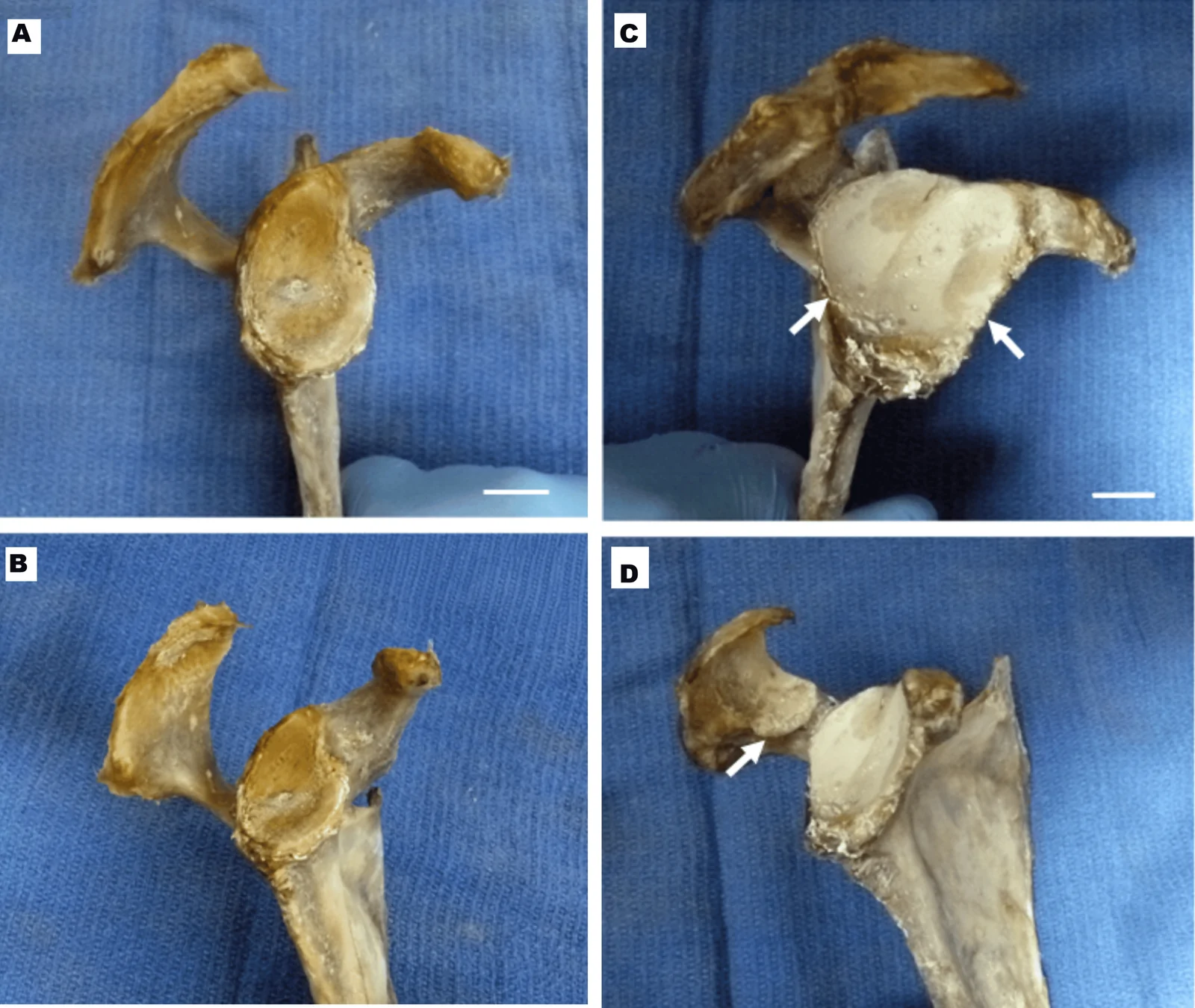
Osseous comparison of the variant right scapula and clavicle with typical specimens after soft-tissue removal. A and B show a typical scapula and clavicle. C and D show the variant specimen from two views; the arrows identify expanded articular regions involving the acromion and the glenoid-adjacent portion of the composite surface. Scale bars represent 1 cm.

The glenohumeral joint capsule was markedly enlarged. From its attachment near the anatomical neck of the humerus, the capsule extended approximately 3 cm inferiorly, forming a blind pouch. It then reflected superiorly to enclose the variant acromial and lateral clavicular components (Figure 1A). This arrangement formed an expanded joint cavity containing the morphologically typical humeral head and the composite concave surface. The gross findings were consistent with a shared capsular space; however, histological examination was not performed, and the tissue covering each component could not be fully characterized.

The infraspinatus and teres minor retained their typical attachments on the middle and inferior facets of the greater tubercle, respectively in addition to these normal attachments, variant fibers from both muscles inserted onto or blended with the posterior aspect of the expanded joint capsule. The tendon of the long head of the biceps brachii was absent from the joint cavity and appeared to originate directly within the intertubercular sulcus. Comparison of the gross findings with typical shoulder anatomy is shown in Table 1.

**Table 1:** Comparison of typical shoulder anatomy with gross findings in the donor *Summarized from Benninger, 2016 [1]. Donor findings are direct gross observations except where "appeared" is used; tissue identity, etiology, and functional consequences were not established.

Feature	Typical anatomy*	Observed anatomy in the donor
Glenohumeral articular surface	The humeral head articulates with the glenoid cavity.	A morphologically typical humeral head opposed a composite concave surface formed by the glenoid cavity, variant acromion, and lateral clavicle.
Glenohumeral and acromioclavicular capsules	The glenohumeral and acromioclavicular articulations have distinct capsules.	On the right, an enlarged capsular space enclosed the glenoid, variant acromial, and lateral clavicular components.
Acromion	The acromion lies outside the glenohumeral capsule and forms part of the superior arch.	The acromion was elongated, contributed to the surface opposing the humeral head, and formed an osseous tunnel near the superior glenoid region.
Lateral clavicle	The lateral clavicle articulates with the acromion and lies outside the glenohumeral capsule.	The lateral end was broad and flattened, with a polished surface positioned to oppose the humeral head.
Supraspinatus	The tendon passes beneath the acromion before inserting on the greater tubercle.	The muscle and tendon passed through the osseous tunnel before reaching the humeral insertion.
Infraspinatus and teres minor	The tendons insert on the middle and inferior facets of the greater tubercle, respectively.	Typical humeral insertions were retained, and variant fibers blended with or inserted onto the expanded posterior capsule.
Long head of the biceps brachii	The tendon typically originates at the supraglenoid region and traverses the joint cavity.	The tendon was absent from the joint cavity and appeared to originate within the intertubercular sulcus.

Measurements of the scapula, acromion, coracoid process, glenoid cavity, and adjacent structures were obtained using digital calipers. Each measurement was obtained three times, and the mean value was recorded. Soft tissue was subsequently removed from selected osseous structures by mechanical dissection followed by immersion in 4% sodium hydroxide, based on the preparation method described by Ajayi et al. [5].

The specimen measurements were compared descriptively with published morphometric data. Acromial length, acromial width, and acromioglenoid distance were compared with measurements reported by Paraskevas et al. [6]. In that study, acromioglenoid distance was defined as the distance between the supraglenoid tubercle and the inferior surface of the acromion, corresponding to the landmarks used in the present case. Maximum acromial length was also compared with direct osteometric data reported by Gallino et al. from 266 scapulae in an Egyptian skeletal collection [7].

Additional direct comparisons were made with measurements reported by von Schroeder et al. [8]. These included the distance from the superior glenoid margin to the scapular notch, lateral thickness of the scapular spine, anteroposterior length and mediolateral width of the acromion, superior glenoid-to-acromial distance, anteroposterior diameter of the glenoid cavity, and coracoid thickness.

North American shoulder measurements reported by Cabezas et al. were included as supplementary population data [9]. Their North American cohort included 92 patients, 90% of whom self-identified as Caucasian, and was evaluated using three-dimensional computed tomography reconstructions. Comparisons were restricted to measurements with sufficiently corresponding anatomical landmarks; non-equivalent measurements were retained only as population context and identified in the table notes. For directly comparable measurements, the percentage difference from each directly comparable published mean was calculated as $\left[\frac{\text{Specimen value} - \text{Published mean}}{\text{Published mean}}\right] \times 100$. Because the studies differed in population, landmark definitions, specimen preparation, and measurement methods, all raw and percentage comparisons were descriptive rather than statistical. The specimen measurements and published comparison data are summarized in Table 2.

**Table 2:** Morphometric findings in the variant right shoulder compared with published data *Cabezas et al. [9] measured lateral distance from the glenoid center to the most lateral point of the acromion; this is not equivalent to the acromioglenoid distance used in the present case. †Cabezas et al. [9] measured superior and inferior glenoid widths separately using three-dimensional computed tomography reconstructions; these values are not directly equivalent to the single anteroposterior glenoid diameter obtained in the present specimen. ‡Gallino et al. [7] reported coracoid-process length and coracoid-base height, not coracoid thickness; those values are therefore not directly comparable with the present specimen measurement. **Values for the variant specimen represent the mean of three repeated digital-caliper measurements. Published values are presented as reported by the respective authors. Percentage difference was calculated only for directly comparable measurements as $\left[\frac{\text{Specimen value} - \text{Published mean}}{\text{Published mean}}\right] \times 100$; positive values indicate a larger specimen measurement and negative values a smaller measurement. The percentages were omitted when the specimen was not measured or the published landmarks were not directly comparable. These comparisons describe magnitude and are not statistical analyses.

Measurement	Variant specimen**	Published comparison data	Descriptive difference from directly comparable mean(s)
Superior glenoid margin to scapular notch	35.99 mm	von Schroeder et al. [8]: 31.8 ± 2.9 mm	+13.2% vs von Schroeder et al. [8]
Lateral scapular-spine thickness	39.67 mm	von Schroeder et al. [8]: 46.1 ± 6.3 mm	-13.9% vs von Schroeder et al. [8]
Acromion anteroposterior length	58.42 mm	Paraskevas et al. [6]: 46.1 mm (range 38-55 mm); von Schroeder et al. [8]: 48.0 ± 5.1 mm; Gallino et al. [7]: 41.5 ± 5.8 mm (range 26.0-56.9 mm). Cabezas et al. [9] did not report a directly comparable measurement	+26.7% [6]; +21.7% [8]; +40.8% [7]
Acromion mediolateral width	28.90 mm	Paraskevas et al. [6]: 22.3 mm (range 19-28 mm); von Schroeder et al. [8]: 21.9 ± 3.7 mm	+29.6% [6]; +32.0% [8]
Maximum anterior acromial thickness	Not measured	Gallino et al. [7]: 6.9 ± 1.6 mm (range 3.0-16.4 mm)	Not applicable
Acromioglenoid distance	35.46 mm	Paraskevas et al. [6]: 17.7 mm (range 13-20 mm); von Schroeder et al. [8]: 15.5 ± 1.8 mm; Cabezas et al. [9]: lateral distance to the acromion, 28.0 ± 4.9 mm*	+100.3% [6]; +128.8% [8]
Glenoid anteroposterior diameter	40.00 mm	von Schroeder et al. [8]: 28.6 ± 3.3 mm; Cabezas et al. [9]: superior width, 23.0 ± 3.0 mm, and inferior width, 28.0 ± 3.2 mm†	+39.9% vs von Schroeder et al. [8]
Glenoid superior-inferior height	Not measured	Cabezas et al. [9]: 35.8 ± 3.5 mm (range 29.3-43.0 mm)	Not applicable
Coracoid thickness	13.05 mm	von Schroeder et al. [8]: 10.6 ± 1.2 mm. Gallino et al. [7] reported coracoid-process length and coracoid-base height, which are not directly comparable‡	+23.1% vs von Schroeder et al. [8]

The acromion had an anteroposterior length of 58.42 mm, compared with 46.1 mm in Paraskevas et al. [6], 48.0 ± 5.1 mm in von Schroeder et al. [8], and 41.5 ± 5.8 mm in Gallino et al. [7]. The specimen measurement exceeded the maximum value of 56.9 mm reported by Gallino et al. Its mediolateral width measured 28.90 mm, compared with 22.3 mm in Paraskevas et al. and 21.9 ± 3.7 mm in von Schroeder et al. [6,8].

The acromion-glenoid distance measured 35.46 mm. Paraskevas et al. reported a mean of 17.7 mm using the same supraglenoid-tubercle and inferior-acromial landmarks [6]. Von Schroeder et al. reported a superior glenoid-to-acromial distance of 15.5 ± 1.8 mm [8]. The specimen value was therefore substantially greater than the means reported in both comparison studies.

The anteroposterior diameter of the glenoid cavity measured 40.0 mm, compared with 28.6 ± 3.3 mm reported by von Schroeder et al. [8]. Cabezas et al. reported superior and inferior glenoid widths of 23.0 ± 3.0 mm and 28.0 ± 3.2 mm, respectively, in their North American cohort [9]. Because the specimen measurement represented a single anteroposterior diameter rather than separate superior and inferior widths, the North American values provide population context but are not directly equivalent.

The distance from the superior glenoid margin to the scapular notch measured 35.99 mm, compared with 31.8 ± 2.9 mm reported by von Schroeder et al.; the lateral thickness of the scapular spine measured 39.67 mm, compared with 46.1 ± 6.3 mm. Coracoid thickness measured 13.05 mm, compared with 10.6 ± 1.2 mm [8].

Every effort was made to follow applicable institutional and international ethical guidance for the use of human cadaveric tissues in anatomical research [10].

## Discussion

For analysis, the findings were separated into: (i) the observed composite anatomical configuration and (ii) changes interpreted as potentially superimposed degeneration. The defining gross configuration consisted of bilateral articulations in which the glenoid cavity, variant acromion, and lateral clavicle collectively formed the surface opposing the corresponding morphologically typical humeral head. In the typical shoulder, the humeral head articulates with the glenoid cavity, while the acromion and lateral clavicle remain outside the glenohumeral joint, and the acromioclavicular articulation is enclosed separately. The present configuration therefore represented a substantial alteration of the observed relationship among the scapula, clavicle, and proximal humerus rather than an isolated variation in acromial shape. Describing this configuration as the principal anatomical finding does not establish whether it was developmental, acquired, or of mixed origin.

The polished lateral clavicular surface was grossly consistent with eburnation, which Prescher described as polished subchondral bone associated with loss of articular cartilage in degenerative joints; they also described acquired grinding facets (facies articularis acromialis) on the inferior acromion resulting from chronic contact with the greater tubercle [11]. These observations are relevant because chronic contact and remodeling could account for some of the articular-appearing surfaces in this donor. However, the present configuration was more extensive than an isolated grinding facet: the acromion and lateral clavicle participated in a composite concave surface within an enlarged capsular space, and comparable structural involvement was present bilaterally. Gross examination supports long-standing mechanical contact but does not establish the tissue composition or whether the underlying architecture was developmental or acquired. Histological sampling of the polished clavicular surface and associated joint tissues would be required to confirm eburnation, pannus, or an articular cartilage remnant.

The morphometric findings support the gross impression of substantially altered superior scapular architecture. Relative to directly comparable published means, the variant acromion was 21.7-40.8% longer and 29.6-32.0% wider, the acromioglenoid distance was 100.3-128.8% greater, and the glenoid anteroposterior diameter was 39.9% greater [6-8]. These percentages quantify the magnitude of difference in this single specimen and do not represent statistical comparisons. The acromial length also exceeded the maximum reported by Gallino et al. [7]. Although Cabezas et al. provided useful North American population context, their computed tomography measurements were not directly equivalent to the osteometric measurements used in this specimen [9]. Because the comparison studies differed in populations, landmark definitions, and measurement methods, the findings should be interpreted descriptively. Collectively, they document an altered spatial relationship among the glenoid, acromion, and clavicle rather than a conventional flat, curved, or hooked acromial morphology [7].

The enlarged joint capsule enclosed the expanded articulation. Its approximately 3-cm inferior blind pouch was larger than the approximately 1-cm inferior axillary recess described by Prescher for the typical glenohumeral capsule [11], although the measurements were not obtained using standardized comparable methods and no percentage difference was calculated. The effect of the altered capsular geometry on humeral translation, stability, or range of motion cannot be determined without ante-mortem imaging or biomechanical assessment.

The variant acromion also formed an osseous arch near the superior glenoid region through which the supraspinatus muscle and tendon passed. This relationship differed from the usual subacromial course of the supraspinatus, but its effect on the tendon space, line of pull, or shoulder function cannot be determined from gross morphology. Although hook-shaped acromial morphology has been associated with subacromial impingement [12], the present configuration should not be classified simply as a hooked acromion because the acromion contributed directly to the composite articulation. In the absence of clinical history, tendon histology, or ante-mortem imaging, impingement or other functional effects cannot be inferred.

The additional fibers from the infraspinatus and teres minor demonstrated that the altered architecture involved the surrounding soft tissues as well as bone. Both muscles retained their typical humeral insertions, but accessory fibers blended with or inserted onto the expanded posterior capsule. Gross examination could not determine the effects of these attachments on capsular tension or force transmission, nor whether they represented primary anatomical variations or secondary adaptations to chronic remodeling.

The long head of the biceps brachii also showed an unusual relationship. The tendon was absent from the joint cavity and originated within the intertubercular sulcus. Chronic degenerative tearing followed by auto-tenodesis within the bicipital groove can produce a similar appearance, but a developmental explanation is also possible. Audenaert et al. described the embryologic migration of the long head of the biceps tendon from an initially extra-articular position toward the supraglenoid region, and extra-articular variants have been interpreted in that developmental context [13]. A comparable groove-origin variant has also been reported in a cadaver [14]. In this donor, the biceps finding therefore remains indeterminate and should not be considered evidence by itself for either a congenital or acquired origin of the composite articulation.

The donor's age also complicates interpretation. Findings treated as potentially degenerative included the inferior glenoid osteophytes, surface irregularities, polished clavicular surface, and proliferative joint soft tissue. Prescher described age-related shoulder osteoarthrosis with osteophytes, cartilage loss, subchondral change, eburnation, and degeneration of the glenoid labrum [11]. These findings were analyzed separately from the composite osseous and capsular configuration and should not be regarded as defining components of its original architecture or evidence of its origin. Without histology, eburnation, pannus, and residual articular cartilage cannot be confirmed. Postmortem change and dissection artifact also cannot be excluded for some soft-tissue irregularities.

Several explanations for the composite articulation remain possible. Its bilateral occurrence raises the possibility of altered joint development, potentially involving abnormal separation or organization of the developing glenohumeral and acromioclavicular regions. Nevertheless, gross dissection alone cannot establish a developmental mechanism. Prescher's discussion of acquired coracoclavicular articulations illustrates that secondary articulations can develop within the shoulder girdle through chronic changes in the relationship of adjacent bones [11]. Trauma, degeneration, or adaptive osseous remodeling could therefore have contributed to the present configuration, and a mixed developmental-degenerative origin cannot be excluded.

The clinical relevance of this variation lies primarily in recognition. An expanded composite articulation involving the glenoid, acromion, and lateral clavicle could be mistaken on imaging or at surgery for severe degenerative disease, post-traumatic deformity, synostosis, or another pathological process. Awareness of such a configuration may therefore be important during radiographic interpretation, arthroscopy, joint injection, distal clavicle procedures, acromioplasty, and rotator cuff surgery. The principal limitations of this report are the absence of ante-mortem imaging, detailed clinical history, histological analysis, and biomechanical testing, as well as less complete documentation of the contralateral shoulder, for which standardized measurements and additional publication-quality photographs were unavailable. Consequently, the developmental origin, tissue composition, and functional consequences of the variation remain uncertain.

## Conclusions

This donor demonstrated bilateral glenohumeral-acromioclavicular composite articulations in which morphologically typical humeral heads opposed surfaces formed by the glenoid cavities, variant acromia, and lateral clavicles. This unusual configuration expands the documented spectrum of shoulder anatomy and may help anatomists, radiologists, and clinicians distinguish anatomical variation from acquired shoulder disease. Its functional consequences and developmental origin remain uncertain in the absence of clinical history, ante-mortem imaging, histological analysis, and biomechanical testing.
